# Neutrophil extracellular traps in autoimmune diseases: Analysis of the knowledge map

**DOI:** 10.3389/fimmu.2023.1095421

**Published:** 2023-01-27

**Authors:** Wei Wang, Jing Su, Wenjuan Kang, Meiqin Yan, Jie Pan, Xianhui Zhang

**Affiliations:** ^1^ Department of Laboratory Medicine, Shanxi Provincial People’s Hospital, Taiyuan, China; ^2^ Department of Internal Medicine, Shanxi Children’s Hospital, Shanxi Maternal and Child Health Hospital, Taiyuan, China; ^3^ Department of Pathology, Stanford University School of Medicine, Palo Alto, CA, United States

**Keywords:** bibliometric analysis, neutrophil extracellular traps, autoimmune disease, systemic lupus erythematosus, rheumatoid arthritis

## Abstract

**Introduction:**

Recent studies have shown much progress in the research of neutrophil extracellular traps (NETs) in autoimmune diseases (AIDs). However, there is no bibliometric analysis in this research field. This study aimed to provide a bibliometrics review of the knowledge structure and research hotspots of NETs in AIDs.

**Methods:**

Articles relevant to NETs in AIDs from 2010 to 2022 were retrieved through the Web of Science Core Collection (WoSCC) database. This bibliometric analysis was performed by VOSview, CiteSpace, and Scimago Graphica.

**Results:**

A total of 289 papers analyzed in this research were from 493 organizations in 47 countries by 1537 authors. They were published in 133 journals and cited 20,180 citations from 2,465 journals. The number of annual publications in this field is growing steadily and rapidly, with the United States, China and Germany leading the research effort. *Frontiers in Immunology and Journal of Immunology* have significantly impacted research in this field. Kaplan, Mariana J, from the National Institutes of Health (The United States), has the most published articles, and Brinkmann, v, from Max Planck Institute for Infection Biology (Germany), is the most co-cited author. Systemic lupus erythematosus and rheumatoid arthritis are the leading topics in this field. The trend of clinical application in the future is the development of new therapies by controlling NETs in the progression of AIDs.

**Conclusions:**

Our study summarized the research trends and developments of NETs in AIDs in recent years and would provide a reference for scholars in this field.

## 1 Introduction

Neutrophils play an essential role in innate immunity by initiating phagocytosis, degranulation and releasing neutrophil extracellular traps (NETs) after recognizing specific pathogens (1, 2). NETs, first reported in 2004, are large reticular structures assembled on a scaffold of decondensed chromatin composed of histones and granular proteins, such as proteinase 3 (PR3), myeloperoxidase (MPO), high mobility group protein B1 (HMGB1), and neutrophil elastase (NE) (3, 4). NETs extrude into the extracellular space primarily through a unique form of cell death known as “NET formation” or NETosis, which allows the neutrophil to effectively capture and kill pathogens at sites of inflammation (5). During infection, NETs can persist for some time, and then be decomposed by the secreted plasma nuclease DNase I or DNase-like protein, thus establishing a delicate balance between the formation and elimination of NETs (6–8). However, tissue damage or autoimmunity may occur when excess NETs yield and dysregulation of suppressive mechanisms (9, 10). Therefore, NETs may be a double-edged sword and are closely associated with the pathogenesis of various diseases (11–18).

Autoimmune diseases (AIDs) are a group of diseases that result in self-tissue damage due to the failure of autoimmune tolerance. The estimated prevalence of AIDs is 7.6–9.4%, including rheumatoid arthritis (RA), systemic lupus erythematosus (SLE), Sjogren’s syndrome (SS), primary biliary cirrhosis (PBC), antiphospholipid syndrome (APS), type 1 diabetes (T1D), autoimmune thyroid disease (AITD) and other nearly 100 different diseases (19–21). In recent years, more and more studies have found the presence of NETs in AIDs. When NETs are overproduced or fail to be cleared, they can deliver multiple autoantigens to the host immune system, inducing autoimmune responses. Therefore, it has been widely accepted that NETs play a crucial role in the pathogenesis and progression of AIDs (9, 11, 12, 22–25).

The bibliometric analysis focuses on studying the publications in a specific research field from the quantitative and qualitative perspectives. Using visualization, the structure, rules and distribution of the knowledge graphs in this research field are presented, and the research status, hotspots and development trends in this field are obtained (26, 27). Currently, the study of NETs in AIDs has not been analyzed by bibliometric methods. To fill this knowledge gap, our study aimed to conduct a bibliometric analysis of NETs in AIDs from 2010 to 2022 to identify the current research status and principal contributors, and to prospect the future research trends in this field.

## 2 Methods

### 2.1 Search strategy

**Figure 1:**
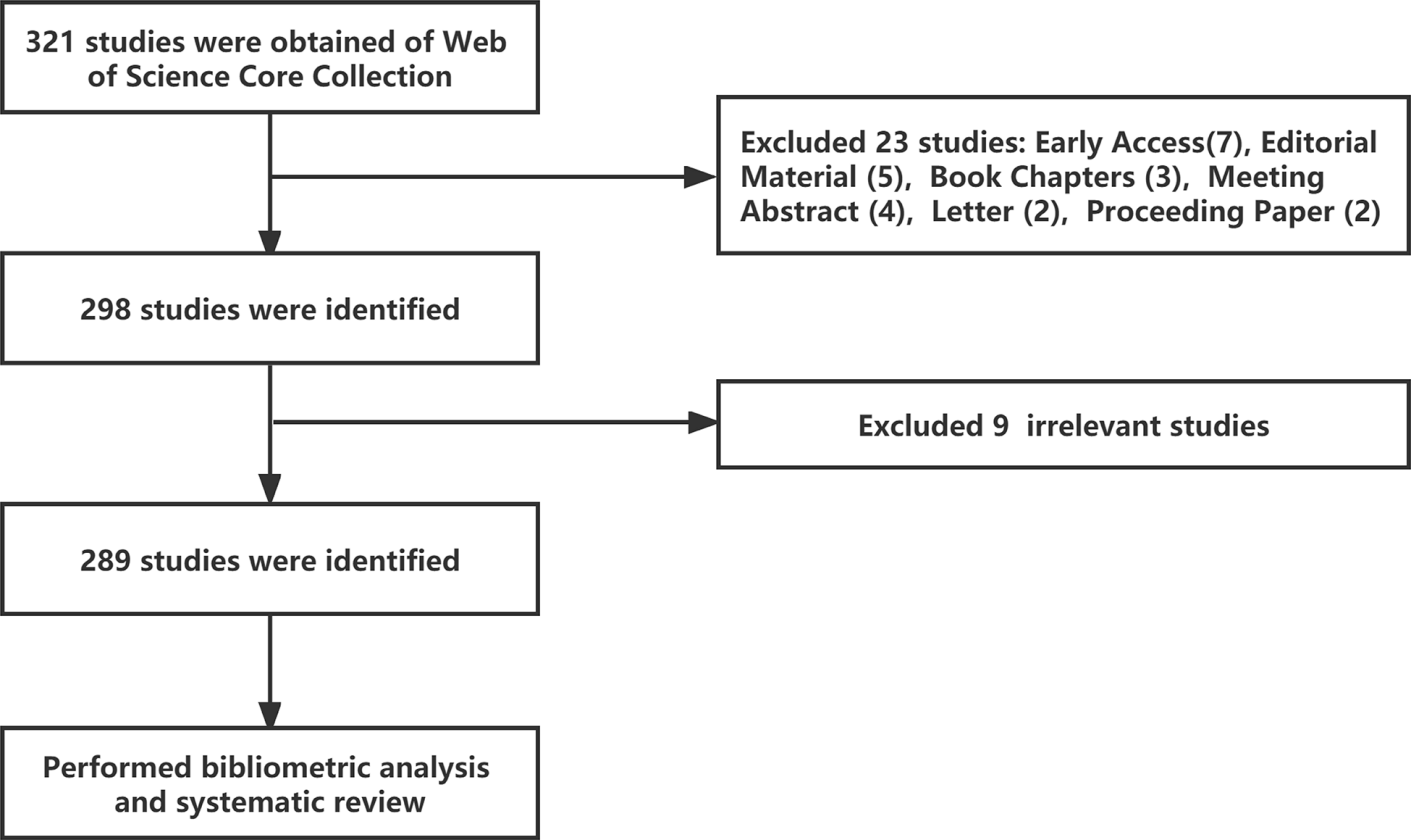
Article selection process for bibliometric analysis and systematic review.

The Web of Science Core Collection (WoSCC) database (https://www.webofscience.com/wos/woscc/basic-search) was selected as the data source. Bibliometric analyses were conducted on December 5, 2022. The chosen search formula was TS= ((“Autoimmune Diseases” or “Rheumatic” or “Rheumatological Diseases”) and (“Neutrophil Extracellular Traps” or “NETs” or “NETosis”)), with a time from 2010-01-01 to 2022-10-31, and 321 literature records were retrieved. Then, literature types were selected as “Article” and “Review Article, and 298 literature records were retrieved in English. Finally, nine irrelevant articles were excluded after the researchers screened the documents, and 289 valid articles were obtained (**Figure 1**).

### 2.2 Data analysis

Bibliometric analysis software VOSviewer (version 1.6.18) and CiteSpace (version 6.1 R2) were used to process and analyze the data obtained. VOSviewer was used to extract key information from publications, and the network view can intuitively reflect the weight of the analyzed data and the relationship between the analyzed data. The different nodes in the map generated by VOSviewer represent items such as countries, organizations, authors, keywords, and the size of the nodes indicate the number of these elements. The lines between nodes reflect the degree of co-operation or co-reference of the project, and are represented by the same color. CiteSpace was used to map the figures for top-level reference bursts, keyword bursts, and keyword time zone maps (26–28). Scimago Graphica (Version 1.0.25) was used to generate a geographic distribution map of publications on NETs in AIDS (29). Microsoft Office Excel 2021 was used to conduct a quantitative analysis of publications.

## 3 Results

### 3.1 Quantitative analysis of publication

**Figure 2:**
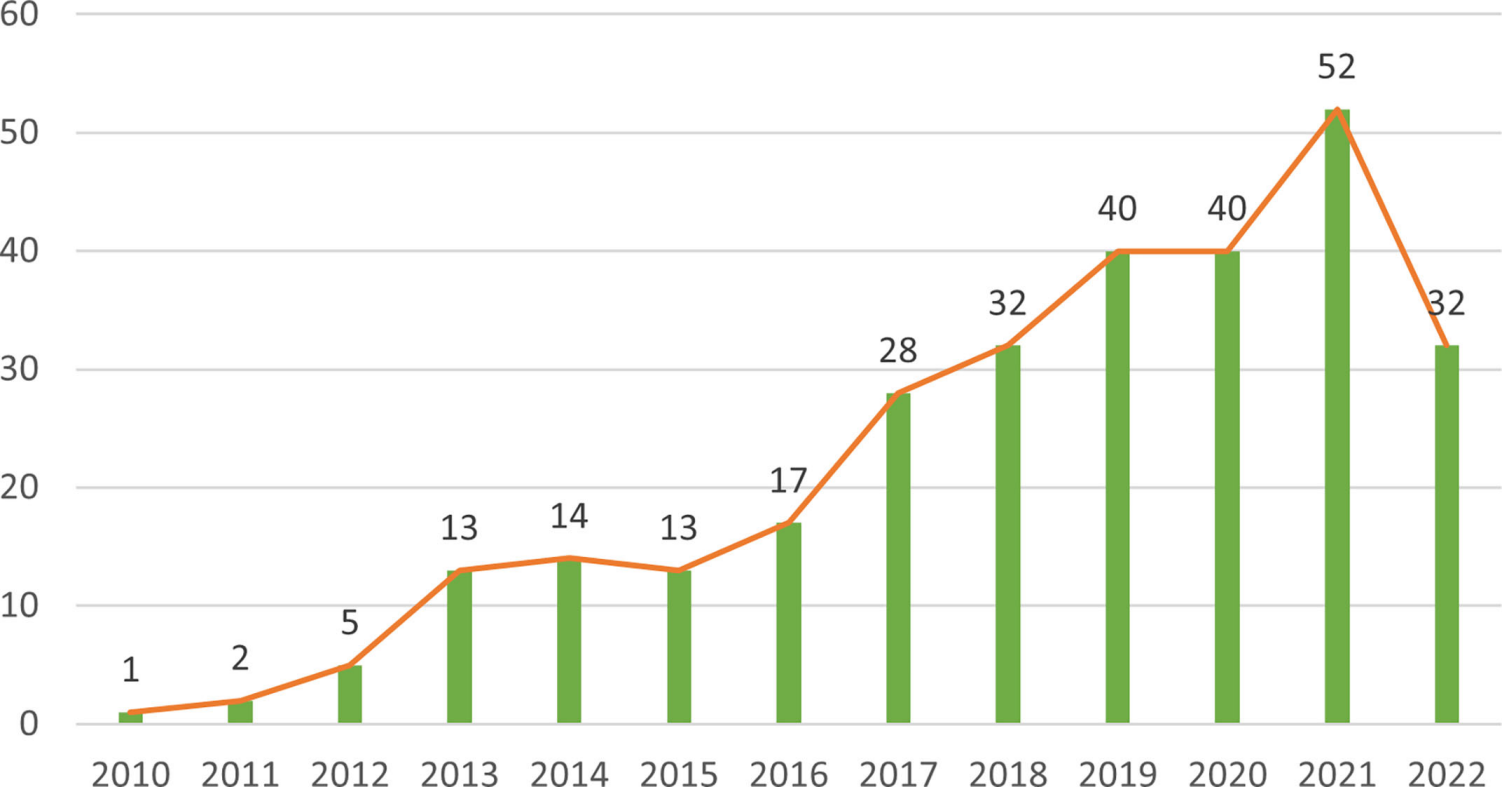
Distribution of publications of NETs in AIDs from 2010 to 2022.

The 289 papers used in this research were from 493 organizations in 47 countries by 1537 authors, published in 133 journals, and cited 20,180 citations from 2,465 journals. **Figure 2** shows the annual number of articles on the research of NETs in AIDs from 2010 to 2022. Overall, annual publications in this field have grown steadily and rapidly. Before 2010, research on NETs in AIDs had not been carried out. From 2010 to 2015, the number of publications was relatively small, and the study was at an early stage. After 2016, the number of publications began to increase significantly. In 2019 and 2020, the number of publications was stable at around 40 per year, and the number in 2021 continued to rise, indicating that NETs have received increasing attention from scholars in recent years and have become a new focus of AIDs research.

### 3.2 Analysis of countries and institutional

**Figure 3:**
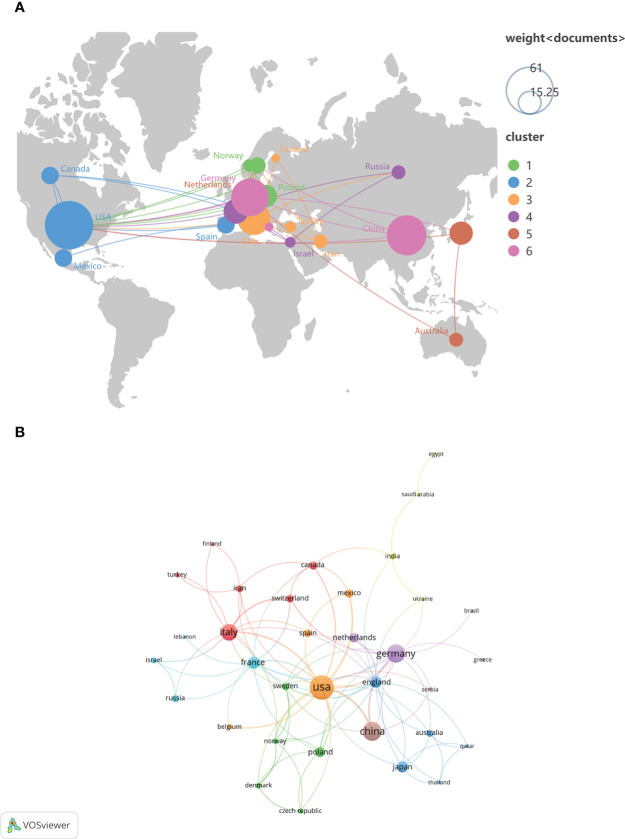
The geographical distribution **(A)** and visualization of countries **(B)** on the research of NETs in AIDs.

To study which countries have made the most outstanding contributions to the NETs research in AIDs, 493 organizations in 47 countries were analyzed. The geographical distribution and visualization of countries are shown in **Figure 3**. The top ten countries with the highest number of publications are distributed in North America, Asia and Europe. The top five countries are the United States (n=70), China (n=43), Germany (n=40), Italy (n=33) and France (n=17). In addition, the average citations/publications are higher in North American and Europe countries and slightly lower in Asia countries (China and Japan) (**Table 1**).

**Table 1:** Top 10 countries and organizations on the research of NETs in AIDs.

Rank	Country	Publications	Citations	Average Citation/Publications	Organization	Publications	Citations
1	The United States (North America)	70	5386	76.94	National Institute of Arthritis and Musculoskeletal and Skin Diseases (The United States)	13	1434
2	China (Asia)	43	866	20.14	University of Erlangen–Nuremberg (Germany)	11	355
3	Germany (Europe)	40	1701	42.53	University of Michigan (The United States)	10	1512
4	Italy (Europe)	33	1407	42.64	Leiden University (Netherlands)	7	356
5	France (Europe)	17	459	27.00	Hokkaido University (Japan)	6	239
6	England (Europe)	15	1970	131.33	University of Washington (The United States)	5	957
7	Japan (Asia)	15	276	18.40	Peking University (China)	4	144
8	Poland (Europe)	13	441	33.92	University of Massachusetts ((The United States)	4	103
9	Netherlands (Europe)	11	490	44.55	National Autonomous University of Mexico (Mexico)	4	64
10	Canada (North America)	9	640	71.11	Shanghai Jiao Tong University (China)	4	49

**Figure 4:**
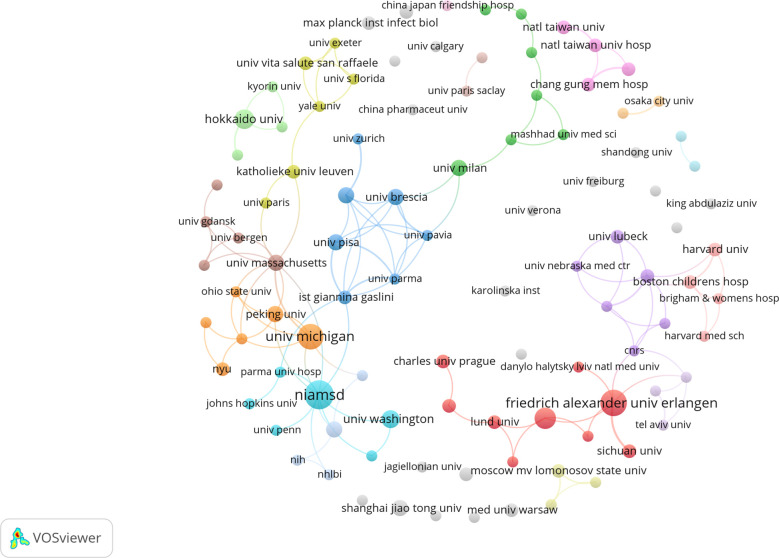
Visualization of organizations related to NETs in AIDs publications.

As shown in **Table 1**, the top ten organizations are in the United States, Germany, Netherlands, Japan, China and Mexico. Subsequently, 102 institutions were selected for a visualization based on a minimum number of publications equal to 2, and a collaborative network was constructed based on each institution’s number and relationship of publications. As shown in **Figure 4**, collaborations mainly occurred among North American and Europe organizations.

### 3.3 Analysis of top journals and co-cited journals

**Figure 5:**
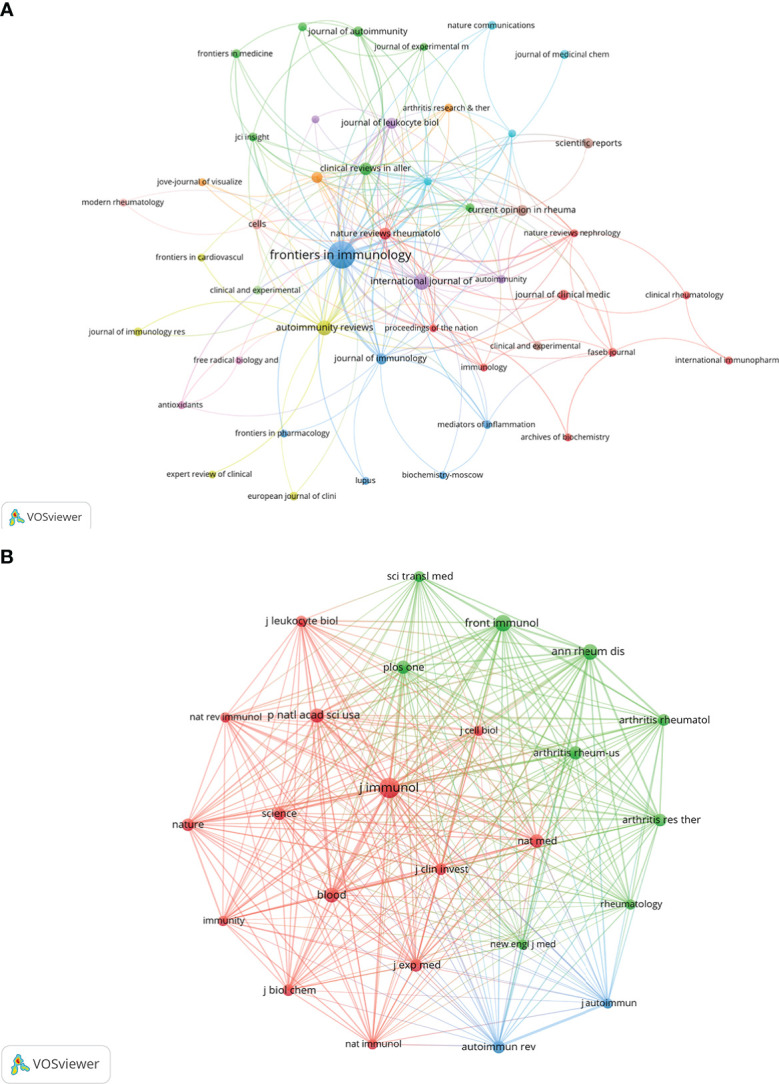
Visualization of journals **(A)** and co-cited journals **(B)** on the research of NETs in AIDs.

Research related to NETs in AIDs was published in 133 journals. The top 15 journals are shown in **Table 2**, with the most published papers being *Frontiers in Immunology* (n=48), followed by *International Journal of Molecular Sciences* (n=12), *Autoimmunity reviews* (n=11), *Clinical reviews in allergy & immunology* (n=7), and *Nature reviews rheumatology* (n=6). In the top 15 journals, the journal with the highest impact factor is *Nature reviews rheumatology* (IF=32.29), followed by *Annals of the rheumatic diseases* (IF=27.97). Then, based on the principle that the minimum number of relevant literatures equals 2, 48 journals were selected and the journal network was drawn (**Figure 5A**).

**Table 2:** Top 15 journals and co-cited journals on the research of NETs in AIDs.

Rank	Journal	Count	IF	Q	Co-cited Journal	Co-citation	IF	Q
1	*Frontiers in immunology*	48	8.79	Q1	*Journal of immunology*	1360	5.43	Q2
2	*International journal of molecular sciences*	12	6.21	Q1	*Frontiers in immunology*	836	8.79	Q1
3	*Autoimmunity reviews*	11	17.39	Q1	*Annals of the rheumatic diseases*	796	27.97	Q1
4	*Clinical reviews in allergy & immunology*	7	10.82	Q1	*Blood*	693	25.48	Q1
5	*Nature reviews rheumatology*	6	32.29	Q1	*Proceedings of the national academy of Sciences of The United States of America*	639	12.78	Q1
6	*Journal of immunology*	5	5.43	Q2	*Arthritis & Rheumatology*	553	15.48	Q1
7	*Journal of leukocyte biology*	5	6.01	Q2	*PLoS One*	547	3.75	Q2
8	*Arthritis & rheumatology*	5	15.48	Q1	*Nature Medicine*	541	87.24	Q1
9	*Current opinion in rheumatology*	5	4.94	Q2	*Science*	492	63.71	Q1
10	*Journal of clinical medicine*	4	4.96	Q2	*Journal of experimental medicine*	490	17.58	Q1
11	*Scientific reports*	4	5.00	Q2	*Autoimmunity reviews*	465	17.39	Q1
12	*Annals of the rheumatic diseases*	3	27.97	Q1	*Arthritis research & therapy*	459	5.61	Q1
13	*Faseb journal*	3	5.83	Q1	*Nature*	434	69.50	Q1
14	*Jci insight*	3	9.48	Q1	*Journal Of Clinical Investigation*	409	19.46	Q1
15	*Nature communications*	3	17.69	Q1	*Journal of biological chemistry*	383	5.49	Q2

On the top 15 co-cited journals, five were cited more than 600 times, with *Journal of Immunology* (Co-citation=1360) cited the most, followed by *Frontiers in immunology* (Co-citation=836), *Annals of the rheumatic diseases* (Co-citation=796), *Blood* (Co-citation=693) and *Proceedings of the national academy of Sciences of The United States of America* (Co-citation=639) (**Table 2**). More than 250 co-citation journals (25 journals) were filtered to produce the top co-cited network (**Figure 5B**). From the analysis results, it can be inferred that *Journal of immunology* and *Frontiers in immunology* have a great influence on the research of NETs in AIDs, and this field is concerned with blood, skin, arthritis and immunology research.

### 3.4 Analysis of top authors and co-cited authors

**Figure 6:**
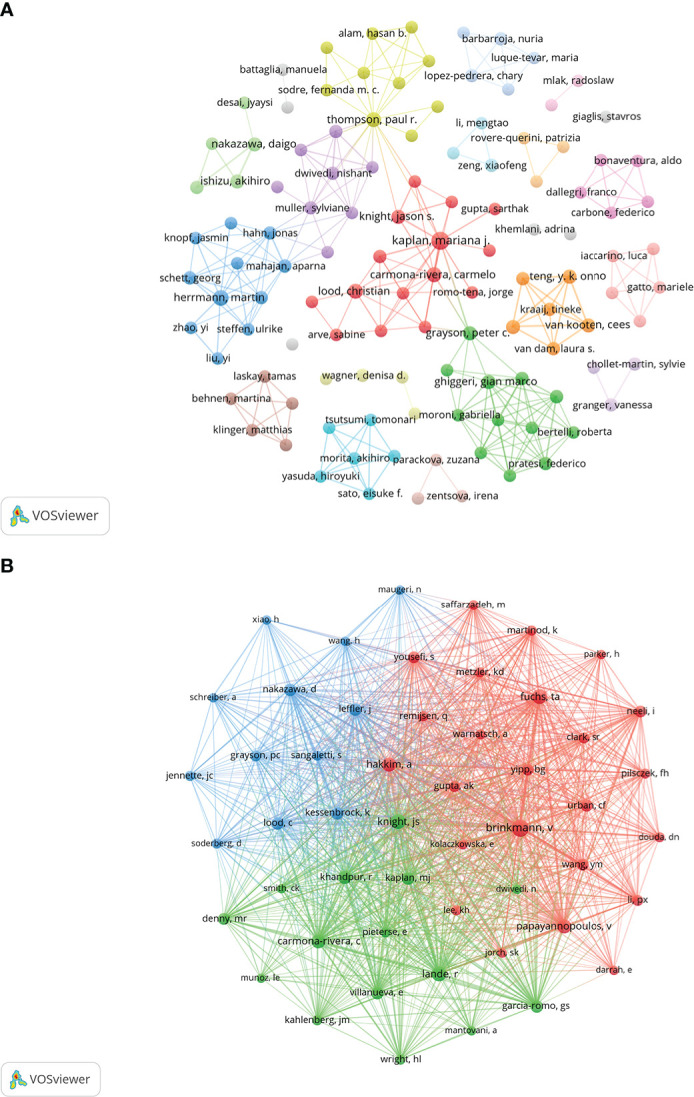
Visualization of authors **(A)** and co-cited Authors **(B)** on the research of NETs in AIDs.

One thousand five hundred thirty-seven authors participated in the published study on NETs in AIDs. The top 10 authors are shown in **Table 3**, and a collaborative network was drawn based on the authors’ published papers (**Figure 6A**). The top five authors with the largest nodes in **Figure 6A**: Kaplan, Mariana J and Carmona-Rivera, Carmelo, from the National Institutes of Health (The United States), focused on identifying mechanisms of immune dysregulation, organ damage, and premature vascular disease in systemic autoimmunity (23, 25, 30–36); Herrmann, Martin, from the University of Erlangen–Nuremberg (Germany), focused on investigating neutrophil-driven inflammation and the role of NETs in this process (6, 37); Thompson, Paul R from UMASS Medical School (The United States) focused on the target-based design of novel anti-cancer and anti-rheumatoid arthritis chemotherapeutics (38, 39).

**Table 3:** Top 10 authors and co-cited authors on the research of NETs in AIDs.

Rank	Authors	Count	Co-cited Authors	Citations
1	Kaplan, Mariana J	18	Brinkmann, v	232
2	Herrmann, Martin	7	Knight, Js	145
3	Carmona-rivera, Carmelo	6	Fuchs, Ta	141
4	Thompson, Paul R	6	Lande, R	126
5	Lood, Christian	6	Hakkim, A	116
6	Nakazawa, Daigo	5	Papayannopoulos, V	113
7	Grayson, Peter c	5	Carmona-Rivera, C	105
8	Knight, Jason s	4	Khandpur, R	89
9	Ihizu, Akihiro	4	Leffler, J	85
10	Rabelink, Ton J	4	GS Garcia-Romo	84

The top 10 co-cited authors were shown in **Table 3**, and 51 (minimum co-citations equal to 30) were filtered to plot the co-citation network (**Figure 6B**). The comprehensive analysis showed that Brinkmann, v, Knight, Js and Fuchs, Ta et al. had an important influence in this field. Brinkmann, v, from Max Planck Institute for Infection Biology (Germany), first reported in 2004 that NETs are an essential step in killing bacteria by neutrophils (3). Knight, Js from the University of Michigan (The United States) was studying the triggers and effectors of autoimmunity in lupus and related antiphospholipid syndrome (APS) (23, 40). The research goal of Fuchs, Ta from University Medical Center Hamburg-Eppendorf (Germany) was to understand how NETs control the progression and outcome of inflammation in different disease categories and to use NETs as a target for developing novel diagnostic assays and therapies for cardiovascular and thromboembolic diseases (8, 41, 42).

### 3.5 Analysis of top co-cited references

**Figure 7:**
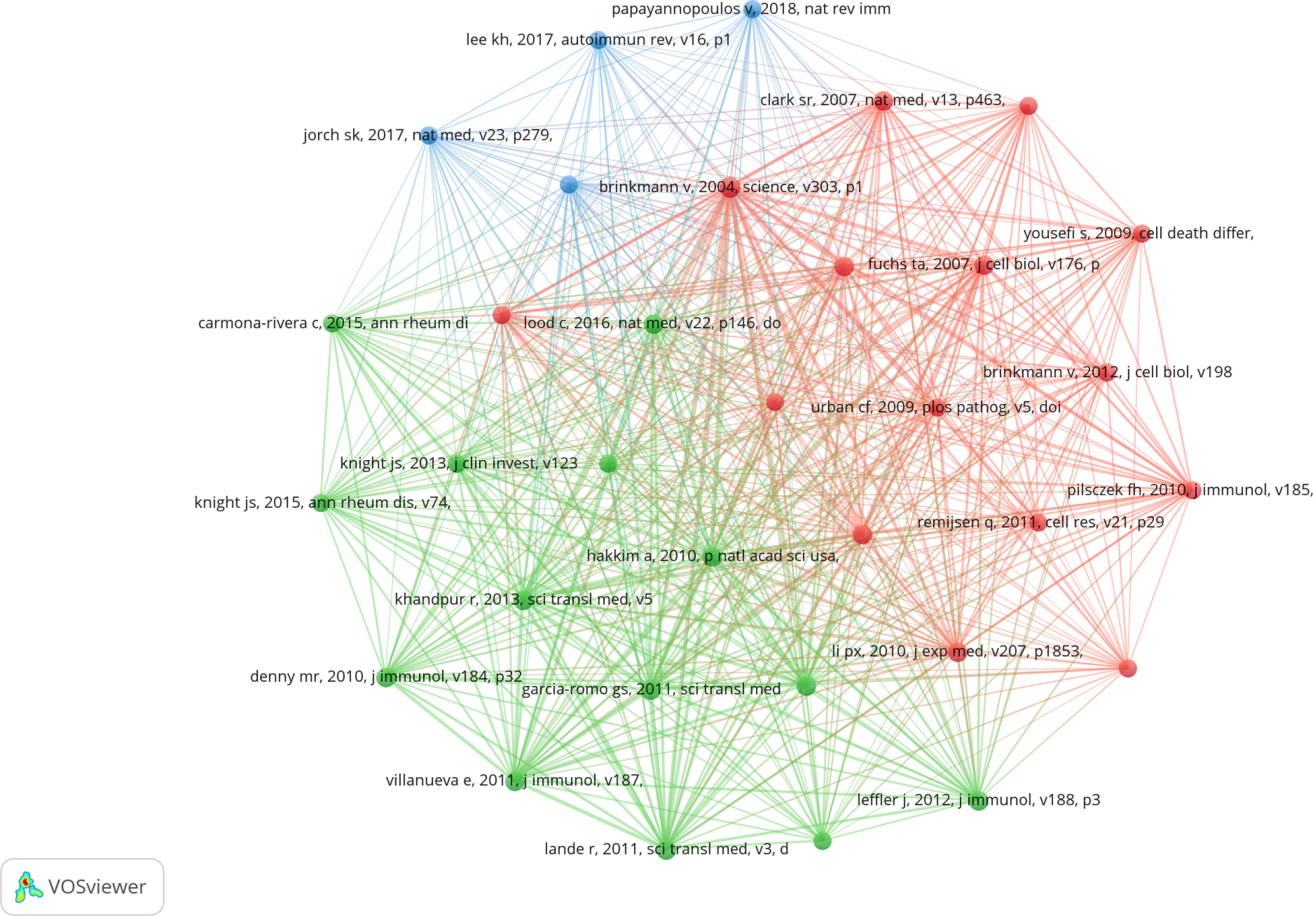
Visualization of co-cited references on the research of exosomes in AIDs.

Co-cited literature refers to literature co-cited by multiple other publications, so that co-cited literature can be regarded as the basis of research in a field (28). From 2010 to 2022, 20,180 references on NETs in AIDs were cited. **Table 4** lists the top 10 references that were co-cited, with all references cited at least 57 times. After filtering out 33 references (the minimum number of co-citations is 30), the co-citation network diagram was drawn (**Figure 7**). The reference “Brinkmann v, 2004, science, v303, p1532, doi 10.1126/science.1092385”, the first reported NETs, was co-cited up to 150 times (3).

**Table 4:** Top 10 co-cited references on the research of NETs in AIDs.

Rank	Co-cited reference	Citations
1	Brinkmann v, 2004, science, v303, p1532, doi 10.1126/science.1092385 (3)	150
2	Hakkim a, 2010, p natl acad sci usa, v107, p9813, doi 10.1073/pnas.0909927107 (6)	85
3	Garcia-romo gs, 2011, sci transl med, v3, doi 10.1126/scitranslmed.3001201 (43)	80
4	Khandpur r, 2013, sci transl med, v5, doi 10.1126/scitranslmed.3005580 (23)	86
5	Lande r, 2011, sci transl med, v3, doi 10.1126/scitranslmed.3001180 (24)	78
6	Kessenbrock k, 2009, nat med, v15, p623, doi 10.1038/nm.1959 (22)	79
7	Villanueva e, 2011, j immunol, v187, p538, doi 10.4049/jimmunol.1100450 (33)	79
8	Fuchs ta, 2007, j cell biol, v176, p231, doi 10.1083/jcb.200606027 (44)	75
9	Lood c, 2016, nat med, v22, p146, doi 10.1038/nm.4027 (45)	60
10	Wang ym, 2009, j cell biol, v184, p205, doi 10.1083/jcb.200806072 (46)	57

### 3.6 Analysis of reference with citation bursts

**Figure 8:**
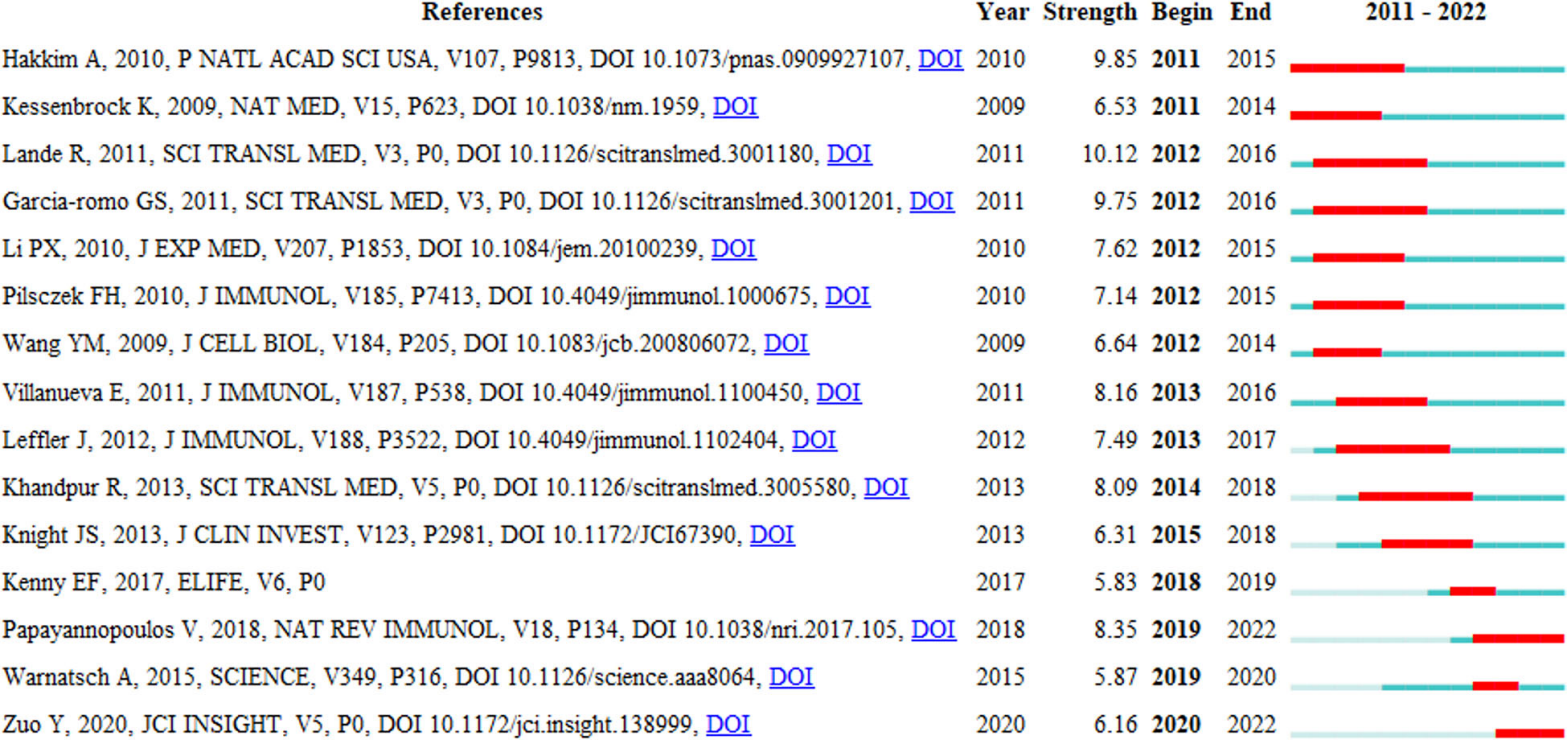
Top 15 references with strong citation bursts. A red bar indicates high citations in that year and the references are ranked by the beginning year of burst.

Reference with Citation Bursts refers to the literature frequently cited by scholars in a particular field over some time. **Figure 8** shows the top 15 references with strong citation bursts (indicated by red bars), and the main research is summarized in **Table 5**.

**Table 5:** The main contents of the 15 references with strong citations bursts.

Rank	Strength	Research Content
1	9.85	Impairment of neutrophil extracellular trap degradation is associated with lupus nephritis (6).
2	6.53	NET formation triggers vasculitis and promotes the autoimmune response against neutrophil components in individuals with autoimmune small-vessel vasculitis (22).
3	10.12	Neutrophils activate plasmacytoid dendritic cells by releasing self-DNA–peptide complexes in systemic lupus erythematosus (24).
4	9.75	Netting neutrophils are major inducers of type I IFN production in pediatric systemic lupus erythematosus (43).
5	7.62	Histone hypercitrullination catalyzed by peptidylarginine deiminase 4 is essential for antibacterial innate immunity mediated by neutrophil extracellular traps (47).
6	7.14	The rapid NET formation has the capacity to ensnare and kill bacteria and perhaps prevent their dissemination (48).
7	6.64	Histone hypercitrullination mediates chromatin decondensation and neutrophil extracellular trap formation (46).
8	8.16	Netting neutrophils induce endothelial damage, infiltrate tissues, and expose immunostimulatory molecules in systemic lupus Erythematosus (33).
9	7.49	Neutrophil extracellular traps that are not degraded in systemic lupus erythematosus activate complement exacerbating the disease (10).
10	8.09	NETs are a source of citrullinated autoantigens and stimulate inflammatory responses in rheumatoid arthritis (23).
11	6.31	Peptidylarginine deiminase inhibition can block NET formation and modulate phenotypes crucial for lupus pathogenesis and disease activity and may represent an important strategy for mitigating cardiovascular risk in lupus patients (49).
12	5.83	NETosis occurs through several signaling mechanisms, suggesting that the extrusion of NETs is important in host defense (50).
13	8.35	Discussed the key findings and concepts that have thus far shaped the field of NET biology (9).
14	5.87	Neutrophil extracellular traps license macrophages for cytokine production in sterile inflammation, such as that seen in atherosclerosis (51).
15	6.16	When not properly regulated, NETs have the potential to propagate inflammation and microvascular thrombosis- including in the lungs of patients with acute respiratory distress syndrome (16).

The citation bursts of references appeared in 2011 at the earliest and 2020 at the latest. Between 2011 and 2018, the citation bursts were mainly related to NETs in SLE, autoimmune small-vessel vasculitis, rheumatoid arthritis and innate immunity. “NET inhibition” was burst cited from 2015 to 2018, and “NETs associated with sterile inflammation and microvascular thrombosis (COVID-19)” were burst cited from 2019 to 2022.

### 3.7 Analysis of hotspots and frontiers

**Figure 9:**
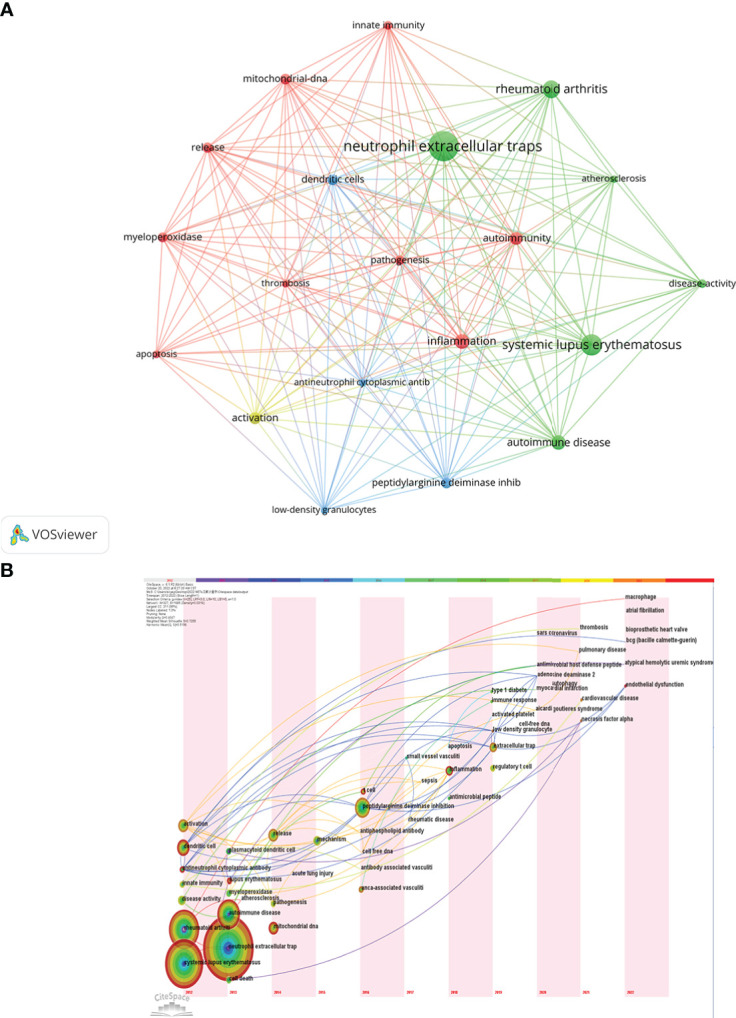
Cluster analysis and trend topic analysis for top 20 keywords **(A)** and Timezone view for keywords **(B)**.

The research hotspots and frontiers of NETs in AIDs could quickly be captured through the co-occurrence analysis of keywords (**Figure 9A**). The top 20 high-frequency keywords are shown in **Table 6**, and the time zone view of keywords was drawn in **Figure 9B**.

**Table 6:** Top 20 keywords on the research of NETs in AIDs.

Rank	Keywords	Counts	Rank	Keywords	Counts
1	neutrophil extracellular traps	160	11	release	23
2	systemic lupus erythematosus	96	12	myeloperoxidase	22
3	rheumatoid arthritis	71	13	pathogenesis	21
4	inflammation	48	14	disease-activity	17
5	autoimmune disease	46	15	innate immunity	17
6	dendritic cells	41	16	apoptosis	15
7	autoimmunity	40	17	antineutrophil cytoplasmic antibodies	14
8	activation	35	18	low-density granulocytes	14
9	mitochondrial-dna	26	19	thrombosis	14
10	peptidylarginine deiminase inhibition	26	20	atherosclerosis	13

Among these keywords, “systemic lupus erythematosus” and “rheumatoid arthritis” appeared more than 70 times, and have always been the high-frequency keywords from 2010 to 2022, representing the main research direction of NETs in AIDs. The keywords “dendritic cells,” “activation,” “mitochondrial-DNA,” and “myeloperoxidase” are related to the role of NETs in pathogenesis. As shown in **Figure 9A**, the keywords in green clusters consist of systemic lupus erythematosus-related and rheumatoid arthritis-related studies, and in red clusters consist of pathogenesis-related studies. Additionally, the keyword “peptidylarginine deiminase inhibition” may be associated with controlling NETs in inflammation progression and uses NETs as a target for developing novel diagnostic assays and therapies. The keyword “atherosclerosis” may be related to the association of NETs with atherosclerosis in patients with autoimmunity, and the keyword “low−density granulocytes (LDGs)” has been implicated in neutrophil subsets of patients with some autoimmune diseases who are more prone to spontaneous release of NETs.

## 4 Discussion

### 4.1 General information

A large number of high-quality studies of NETs in AIDs have been conducted. From 2010 to 2015, research in this area was still in its infancy; after 2016, the publications began to increase significantly. In 2019 and 2020, the number of articles published was stable at about 40 per year, and continued to rise in 2021. In recent years, NETs have become a new focus of research in AIDs, and annual publications in this field are growing steadily and rapidly. The United States, China, Germany, Italy and France are the leading countries to carry out NETs research in AIDS. The top ten organizations are in the United States, Germany, Netherlands, Japan, China and Mexico. In addition, the average citations/publications are higher in North America and Europe, which may be related to the earlier initiation of research in this field in the relevant countries.

The journals *Journal of immunology* (IF=5.43) and *Frontiers in immunology* (IF=8.79) have a significant influence on the research of NETs in AIDs. *Frontier in immunology* published the most articles in the field of NETs in AIDs (n=48), and *Journal of Immunology* (Co-citation=1360) was cited the most. Moreover, the journal with the highest impact factor is *Nature reviews rheumatology* (IF=32.29). From the authors’ perspective, Kaplan, Mariana J and Carmona-Rivera, Carmelo from the National Institutes of Health published the most articles. They focused on the mechanisms of immune dysregulation, organ damage, and early-onset vascular disease in AIDs, and have proposed that NET formation and its subsequent responses play an essential role in lupus pathogenesis by damaging endothelial cells and synthesizing elevated levels of proinflammatory cytokines and type I IFNs (33–35). Furthermore, they propose that NETosis in RA pathogenesis is accelerated by the externalization of citrullinated self-antigens and immunostimulatory molecules, which may promote aberrant adaptive and innate immune responses in joints and the periphery, and perpetuate the pathogenic mechanism of the disease (23, 36). In terms of co-cited authors, Brinkmann first reported in 2004 that NETs are an important step in neutrophil bactericidal action with essential implications in this field (2).

### 4.2 Research basis

Through co-citation analysis, we can understand the basis of research in the field of NETs in AIDs (28). NETs, first reported in 2004, extruded into the extracellular space primarily through a unique form of cell death known as NETosis, enabling the neutrophil to potently capture and kill pathogens at sites of inflammation (3, 5). In recent years, several different NETs formation pathways have been revealed. The most common pathway is the traditional suicidal NETosis, also known as the reactive oxygen species (ROS)-dependent pathway. NETosis is initiated when neutrophils are exposed to various stimuli (extracellular microbes or ribonucleoprotein immune complexes), activating the Raf/MERK/ERK signal transduction pathway *via* protein kinase C. Subsequently, the levels of reactive oxygen species (ROS) are elevated by activated NADPH oxidase complex (NOX), promoting the degradation of neutrophil elastase (NE) and cytoplasmic granules containing myeloperoxidase (MPO). NE and MPO, together with peptidyl arginine deiminase 4 (PAD4), induce the citrullination of histone H3 (CitH3), further leading to chromatin decondensation. NETs are formed by the combination of chromatin and granule proteins and are released into the extracellular space once the cell membrane ruptures, ultimately leading to neutrophil death (5, 9, 48, 52–54). Another pathway is the ROS-independent pathway, in which neutrophils release NETs without disrupting the nuclear or plasma membrane, and retain several conventional functions. After microbial products or activated platelets are recognized by Toll-like receptors or C3 complement receptors on neutrophil cell membranes, the resulting cytosolic calcium peak activates PAD4, triggering chromatin decondensation, and nuclear DNA is then packaged into vesicles and released in the form of budding. Neutrophils released by NETs in this way can still perform cellular functions, such as cell phagocytosis and migration (4, 46, 55). Another ROS-dependent form of mitochondrial NETosis, which releases mitochondrial DNA rather than nuclear DNA, has also been reported. After stimulation with lipopolysaccharide (LPS) or C5a, mitochondria in neutrophils serve as a ROS generator and facilitate the innate immune function of neutrophils *via* NADPH oxidase-independent NET formation, which is not accompanied by neutrophil death (45, 56, 57). Notably, different stimuli appear to activate different pathways of NET formation, and the role of these pathways in neutrophil dysregulation and autoimmunity has not been determined systematically.

### 4.3 Hotspots and frontiers

Analysis of references with citation bursts and keywords can quickly grasp the hotspots of distribution and evolution in the field of NETs in AIDs. At present, systemic lupus erythematosus and rheumatoid arthritis are the main research topics on NETs in AIDs. In addition, developing new therapies by controlling the progress of NETs is the trend of clinical application in the future.

#### 4.3.1 Neutrophil extracellular traps in systemic lupus erythematosus

Systemic lupus erythematosus (SLE) is a chronic autoimmune disease affecting multiple organs, such as the skin, kidneys, and blood vessels. It is characterized by the overproduction of autoantibodies against nuclear antigens (nucleic acids and associated proteins). The autoantibodies and immune complexes can induce NET formation, and subsequently activate plasmacytoid dendritic cells (pDCs) to produce type I interferon (24, 43, 45). In addition, a neutrophil population called low-density granulocytes (LDGs) is elevated in SLE and shows an increased capacity to release NETs (34). Moreover, owing to the presence of DNase I inhibitors or autoantibodies against NETs that block the access of DNase I into NETs, patients with SLE exhibit a reduced ability to degrade NETs (6, 58).

Excessive NET formation and impaired clearance contribute to the persistence and prolongation of NETs in SLE patients. The elevated autoantigens and immunostimulatory proteins, such as IL−17 and IL−37 in NETs, can promote the secretion of type I IFN by activating TLR9 in plasmacytoid dendritic cells. And it can also facilitate the activation of NLRP3 inflammasome in macrophages, thus improving the levels of active IL−1β and IL−18 to promote the NETs release, further amplifying the inflammatory responses. In turn, the active IL-18 can induce NETosis, creating a proinflammatory feedback loop (24, 30). In addition, increasing the expression level of matrix metalloproteinase-9 (MMP-9) in NETs induces endothelium apoptosis, thereby activating endothelial MMP-2 (25). Moreover, the components of NETs can mediate the oxidation of high−density lipoprotein, resulting in SLE-related atherosclerotic cardiovascular disease (59).

#### 4.3.2 Neutrophil extracellular traps in rheumatoid arthritis

Rheumatoid arthritis (RA) is a chronic systemic autoimmune disease that affects joints and connective tissue, and is characterized by the production of autoantibodies against citrullinated protein antigens (ACPAs). Like SLE, NETs are closely associated with the pathogenesis of RA, and the formation of NETs is increased in synovial and peripheral blood neutrophils (60). Concomitant with NETs formation in RA patients, histones and other NET-associated proteins can be citrullinated by PAD enzymes (in particular PAD4), and citrullinated NETs proteins are important autoantigens in RA (61).

NETs released by neutrophils in RA patients are more numerous and faster than in healthy subjects. NETs can promote the production of ACPAs, release immune stimulatory molecules such as IL-6 and IL-8, and stimulate autoimmune responses. In turn, ACPAs can also stimulate the production of NETs, thus forming a vicious cycle. NETs levels in RA patients were positively correlated with ACPAs levels (22, 58). IL6 may induce B cells to mature plasma cells and indirectly promote the formation of NETs by generating ACPAs. NETs also release carbamylated proteins (CarP), leading to the production of CarP antibodies and carbamylated NETs protein antibodies, which activate macrophages to release proinflammatory cytokines and stimulate fibroblast-like synoviocyte (FLS) to release RANKL to promote osteoclast formation and activation (62). Moreover, neutrophil elastase (NE) in NETs can directly degrade cartilage components, and amplified FLS and macrophage can further contribute to joint injury (63).

#### 4.3.3 Neutrophil extracellular traps in other autoimmune diseases

In addition to SLE and RA, NETs have also been revealed in other AIDs. Antiphospholipid antibodies (aPLs) purified from the patients of antiphospholipid syndrome (APS) can induce NETs formation in neutrophils of healthy volunteers, and therefore the induction of NETs by aPLs may be one of the reasons for APS (40). On the other hand, the degradation of NETs in the serum of APS patients is impaired, which is closely related to the specific clinical manifestations and antibodies against NETs in patients with secondary APS (64). In the patients of anti-neutrophil cytoplasmic antibody (ANCA) associated vasculitis (AAV), the serum DNase1 activity and the ability to degrade NETs are significantly lower compared to healthy individuals, and excessive NETosis can lead to complement generation and endothelial damage (65, 66). In the patients of Behcet’s disease (BD), circulating neutrophils are more likely to release NETs *in vitro* and express higher levels of PAD4 than healthy control. In addition, in biopsies of vasculitis from BD patients, NETosis is found mainly around the affected blood vessels. These results suggest that NETosis plays a role in the pathophysiology of BD, especially in the induction of vasculitis (67, 68). Together, these findings further clarify the critical role of NETs in the pathogenesis and development of AIDs.

#### 4.3.4 NETs as a target to develop therapies

Given the importance of NETs in AIDs, developing novel therapies targeting the formation or clearance of NETs might be a potential clinical strategy (54). DNAse is most commonly used to dismantle DNA, the main framework of NETs, which could degrade NETs and apoptotic cell microparticles and suppresses autoreactivity, thus having therapeutic effects in SLE and lupus nephritis (69, 70). Some stimuli can bind to neutrophil receptors, such as TLRs and complement receptors, to activate neutrophils and trigger NETosis. Disruption of these interactions can inhibit the NETosis process and disease progression. Hydroxychloroquine (HDQ) is an antimalarial drug that could reduce NETosis by inhibiting the expression of PAD4 and Rac2, as well as inhibiting TLR9. And TAK-242 is also a TLR4 inhibitor that could reduce NET formation, suggesting that it may be helpful in treating AIDS (69, 71). NETosis requires calcium mobilization. Therefore, calcineurin inhibitors such as cyclosporine A or tacrolimus would be potential therapeutic agents to inhibit the calcineurin pathway, and reduce the activation of neutrophils in AIDs. In fact, they have been used to treat patients with SLE and have been very productive (69, 72). ROS is essential for forming NETs, and a series of ROS scavengers have therapeutic effects on AIDs. For example, ROS scavengers such as N−acetylcysteine (NAC) could suppress ROS production to reduce NETosis, and the application of NAC can improve disease outcomes in SLE patients (73). Citrullination of histones is associated with chromatin decondensation during the formation of NETs, and thus PAD inhibitors are thought to prevent NETs formation and have also been studied in AIDs. PAD inhibitors were observed to reduce NETs formation effectively and disease severity in mouse models of SLE and RA (74, 75). Vitamin D can inhibit the adaptive immune system (76), and it has been reported that Vitamin D 1,25(OH)2D3 can inhibit NETs-induced endothelial damage in SLE patients by inhibiting NE externalization (77).

Although it is feasible to target NETs for the therapeutic benefit of AIDs in clinical practice, some scholars believe that neutrophilic phagocytosis plays a key role in host defense, and any therapy targeting NETs formation must avoid impairing the physiological function of these cells. Meraj A et al. have suppressed both types of NETosis by inhibiting transcription, while maintaining the production of ROS, so that the function of physiological neutrophils would not be lost (78). Therefore, it is a promising approach to use a NETs formation inhibitor that selectively impinges NETs production while retaining phagocytosis and degranulation in the future.

### 4.4 Advantages and shortcomings

Our visualization analysis could provide more insight into hotspots and frontiers than traditional reviews. We comprehensively analyzed the research trends and developments of NETs in AIDs by bibliometrics for the first time, and would provide a reference for scholars in this field.

Limited by the data extracted only from the WoSCC database, some relevant studies might be missed. Second, we filtered data published in English, and the trends of non-English writing papers were ignored. Moreover, bias could also come from the data extraction protocol.

### 4.5 Conclusion

NETs played a crucial role in the pathogenesis and development of AIDs, and annual publications in this field are growing steadily and rapidly. The United States, China and Germany are major countries to carry out the related research, and *Frontiers in Immunology* and *Journal of Immunology* have a significant influence on the research. Systemic lupus erythematosus and rheumatoid arthritis are the current major topics in this field, and the development of new treatment methods *via* control of NETs in the progression of AIDs is the trend of future clinical application. Drugs targeting NETs formation have shown promise in clinical models and are an exciting possibility for treating AIDs.

## Data availability statement

The original contributions presented in the study are included in the article/supplementary material. Further inquiries can be directed to the corresponding author.

## Author contributions

WW and XZ contributed to conception and design, and initial draft of the manuscript. XZ, MY, and JS contributed to literature search, analysis and drew pictures. JP contributed to edited drafts of the manuscript. JS reviewed and revised the manuscript. All authors contributed to the article and approved the submitted version.
