# Corrigendum: Neutrophil extracellular traps in autoimmune diseases: Analysis of the knowledge map

**DOI:** 10.3389/fimmu.2023.1158560

**Published:** 2023-02-22

**Authors:** Wei Wang, Jing Su, Wenjuan Kang, Meiqin Yan, Jie Pan, Xianhui Zhang

**Affiliations:** ^1^ Department of Laboratory Medicine, Shanxi Provincial People’s Hospital, Taiyuan, China; ^2^ Department of Internal Medicine, Shanxi Children’s Hospital, Shanxi Maternal and Child Health Hospital, Taiyuan, China; ^3^ Department of Pathology, Stanford University School of Medicine, Palo Alto, CA, United States

**Keywords:** bibliometric analysis, neutrophil extracellular traps, autoimmune disease, systemic lupus erythematosus, rheumatoid arthritis

In the published article, there was an error in the author list, and author Wenjuan Kang was erroneously excluded. Additionally, author Jing Su was changed to a corresponding author. The corrected author list appears below.

Wei Wang^1^, Jing Su^2^* ^†^ , Wenjuan Kang^2^, Meiqin Yan^2^, Jie Pan,^3^ Xianhui Zhang^2^* ^†^

† These authors have contributed equally to this work

In the published article, there was an error. Instead of “exosomes”, it should be “neutrophil extracellular traps (NETs)”.

A correction has been made to **Abstract**, *Introduction*.

This sentence previously stated:

“Recent studies have shown much progress in the research of exosomes in AIDs. However, there is no bibliometric analysis in this research field. This study aimed to provide a bibliometrics review of the knowledge structure and research hotspots of neutrophil extracellular traps (NETs) in autoimmune diseases (AIDs).”

The corrected sentence appears below:

“Recent studies have shown much progress in the research of neutrophil extracellular traps (NETs) in autoimmune diseases (AIDs). However, there is no bibliometric analysis in this research field. This study aimed to provide a bibliometrics review of the knowledge structure and research hotspots of NETs in AIDs.”

The authors apologize for these errors and state that they do not change the scientific conclusions of the article in any way. The original article has been updated.

